# Impact of Ultrasonic Activation on the Effectiveness of Sodium Hypochlorite: A Review

**DOI:** 10.7508/iej.2015.04.001

**Published:** 2015

**Authors:** Zahed Mohammadi, Sousan Shalavi, Luciano Giardino, Flavio Palazzi, Saeed Asgary

**Affiliations:** a *Iranian Center for Endodontic Research, Research Institute of Dental Sciences, Dental School, Shahid Beheshti University of Medical Sciences, Tehran, Iran; *; b * Iranian National Elites Foundation, Tehran, Iran; *; c * Private Practitioner, Hamedan, Iran****; ***; d *Department of Periodontology, Endodontology, Pharmacology and Microbiology, Dental School, University of Brescia, Italy****; ***; e *Department of Odontostomatological and Maxillofacial Sciences, Federico II University of Naples, Italy*

**Keywords:** Antibacterial Activity, Biofilm, Root Canal Irrigants, Smear Layer, Sodium Hypochlorite, Ultrasonic Activation

## Abstract

Using ultrasonic devices in endodontics can enhance the antibacterial and tissue dissolving ability of different root canal irrigants such as sodium hypochlorite (NaOCl) which is the most common irrigant with excellent antibacterial and tissue dissolving abilities. However, due to its high surface tension, its penetration into the irregularities of the root canal system is a challenge. The purpose of this paper was to review the different ultrasonic devices, different types of ultrasonic irrigation, the effect(s) of ultrasonic activation on the antibacterial and biofilm-removal abilities of NaOCl as well as the effect of ultrasonic activation on the smear layer removal ability of NaOCl.

## Introduction

Due to the complex anatomy of the root canal system, and presence of intra-canal irregularities such as oval extensions, isthmi and apical deltas, it is difficult if not impossible to sterile the root canal system [1]. According to Wu *et al.* [2], only 40% of the root canal walls in the apical area of oval canals can be contacted by rotating instruments. Therefore, irrigation and chemical debridement are essential parts of root canal treatment as it allows for cleaning beyond the root canal instruments [3, 4]. 

The aim of root canal irrigation is to remove the pulp tissue remnants and microorganisms (in either planktonic or biofilm forms) [5], eliminate the smear layer (SL) and extirpation of dentine debris during root canal treatment [6]. Sodium hypochlorite (NaOCl) is the most common root canal irrigation solution. One of the major drawbacks of NaOCl is the high surface tension, which affects the tubular penetration and thus antibacterial ability of NaOCl [7]. In the absence of cementum and during a three-week incubation, *Enterococcus faecalis *(*E. faecalis*) can penetrate the dentinal tubules of root canal walls up to 800-1000 µm deep [8], whereas the maximum depth of 6% NaOCl penetration into the dentinal tubules is reported to be 300 µm after 20 min at 45^°^C [9].

***Active and passive root canal irrigation***

*Passive* irrigation is conducted by slow dispensing of the irrigant of choice into a canal through a variety of different gauged needles [10]. In order to allow the irrigant to reflux and move the debris coronally, the needle should be loose in the canal. To achieve deeper and more effective placement, smaller gauged needles should be chosen [11]. Passive irrigation has limitations because the static reservoir of irrigant restricts the penetration, circulation and cleansing potential of the irrigation solution of a root canal system [11].

On the other hand, *active* irrigation initiates dynamics and flow within the fluid and thus improves root canal disinfection. In well-shaped canals, fluid activation has a critical role in cleaning and disinfection of the canal irregularities by facilitating the fluid penetration through all aspects of the root canal system [2, 11]. 

***Physics of ultrasonic***

Ultrasound is a vibration or acoustic wave with similar nature as sound but with a frequency higher than the highest frequency detectable by the human ear (approximately 20000 Hz) [12]. Ultrasonic tips have an important advantage over hand and rotary instruments because they do not rotate, thereby deliver safety and control while maintaining high cutting efficacy [13]. 

There are two basic methods for producing an ultrasonic wave. First is *magnetostriction* that converts the electromagnetic energy into mechanical energy. The second method works according to the *piezoelectric* principle and uses a crystal which changes in size by applying electrical charge [14, 15]. Therefore, without producing heat, the crystal undergoes mechanical oscillation. 

Magnetostrictive units have two major drawbacks for endodontic application. First they have elliptical movement and oscillate in a figure-eight manner and second, they generate heat, so adequate cooling is required.

One major advantage of piezoelectric units over magnetostrictive devices are production of more cycles per second (40 in piezoelectric* vs.* 24 in magnetostrictive devices). The other advantage is the piston-like linear movement of tip in piezoelectric units from back to front which is ideal for endodontic treatment [16, 17].

## Materials and Methods

***Retrieval of literature***

An English-limited Medline search was performed through the articles published from 1980 to 2014. The searched keywords included “Ultrasonics AND Sodium Hypochlorite”, “Ultrasonics Activation AND Sodium Hypochlorite", "Ultrasonic AND NaOCl”, “Passive Ultrasonic Activation AND Sodium Hypochlorite”. Then, a hand search was done in the references of result articles to find more matching papers.

## Results

A total of 225 articles were found which in order of their related keywords are “Ultrasonics AND Sodium Hypochlorite (103 articles)”, “Ultrasonics Activation AND Sodium Hypochlorite (47 articles)", "Ultrasonics AND NaOCl (51 articles)”, “Passive Ultrasonic Activation AND Sodium Hypochlorite (24 articles)”.

## Discussion

***Effects of ultrasonic irrigation in endodontics***

Using ultrasonic energy in endodontic treatment has improved treatment quality in many aspects, including access to root canal entry holes, cleaning, shaping and filling the canals, eliminating the obstructions and intracanal materials and endodontic surgery [17]. 

Ultrasonic devices can be utilized in two manners; simultaneous combination of ultrasonic irrigation/instrumentation and passive ultrasonic irrigation (PUI) [16, 18]. Because of the difficulty in controlling dentin removal and subsequently the final shape of the canal, the first method is almost discarded in the clinical practice. Ultrasonic energy cannot be considered as an alternative to conventional manual instrumentation [1, 18, 19]. 

Applying ultrasound for passive irrigation seems more advantageous [20, 21]. For the first time, the term PUI was proposed to describe irrigation without simultaneous instrumentation. This reduces the rate of potential endodontic mishaps in the root canal system. During this process, energy is transmitted from a file or smooth oscillating wire to the irrigant by means of ultrasonic waves and creates streaming and cavitation within the irrigant solution [18].

***Effect of ultrasonic energy on antibacterial activity of NaOCl***

NaOCl is the most common root canal irrigant with excellent antibacterial and tissue dissolving abilities [22]. Irrigation with NaOCl combined with ultrasound or a wave vibration system has the greatest antibacterial effect. This combined method improves the exchange of substances in the canal, permits heating of the irrigating substance, and eliminates dentin debris and the waste layer, thereby achieving greater cleaning effect [23]. In general, the literature recommends 30 sec to 3 min being dedicated to NaOCl irrigation, although there is no defined consensus on the exact duration of time. Shorter passive irrigation makes it easier to keep the file in the center of the canal [20].

In an *in vitro* study by Tardivo *et al.* [24] there was no significant difference between PUI, syringe irrigation and passive sonic activation in eliminating *E. faecalis*. Huque *et al.* [25] showed the superiority of PUI over syringe irrigation. On the other hand, Alves *et al.* [26] and Siqueira *et al.* [27] have indicated no significant difference between PUI and syringe irrigation.

***Ultrasonics and bacterial biofilms***

According to Bhuva *et al.* [28] both conventional syringe irrigation and PUI with 1% NaOCl were effective at complete removal of the intra-radicular *E. faecalis* biofilms. Harrison *et al.* [29] concluded that after canal preparation in straight root canals PUI for 1 min with 1% NaOCl is potentially an effective supplementary step in microbial control.

Bhardwaj *et al.* [30] showed that 1% NaOCl with PUI could effectively in remove *E. faecalis* biofilm. Neelakantan *et al.* [31] showed that laser activation of NaOCl was more effective against *E. faecalis* biofilm compared to the ultrasonic. 

***Effect of ultrasonic on smear layer removal***

Ahmad *et al.* [32] claimed that modified ultrasonic instrumentation using 1% NaOCl removed the debris and smear layer very effectively. However, Martin and Cunningham [33] showed that ultrasonic activation of NaOCl was not effective in removing the smear layer. The apical region of the canals showed less debris and smear layer than the coronal aspects, depending on acoustic streaming, which was more intense in magnitude and velocity at the apical segments of the file. Cameron [34] also compared the effect of different ultrasonic irrigation periods on removing the smear layer and found that 3 and 5-min irrigation produced smear-free canal walls, whilst 1-min irrigation was ineffective. In contrast to these results, other investigators found ultrasonic preparation unable to remove the smear layer [35-37].

Researchers who found the cleaning effects of ultrasonic beneficial, used the technique only for the final irrigation of root canal after completion of hand instrumentation [32, 38, 39]. Ahmad *et al.* [32, 40] claimed that direct physical contact of the file with the canal walls throughout instrumentation reduced acoustic streaming. Acoustic streaming is maximized when the tips of the smaller instruments vibrate freely in a solution. Lumley *et al.* [41] recommended that only #15 files must be used to maximize the micro-streaming effect for the removal of debris.

Prati *et al.* [42] also mentioned smear layer removal with ultrasonics. Walker and del Rio [43, 44] showed no significant difference between tap water and NaOCl when used with ultrasonication; however, neither solution was effective at any level in the canal to remove the smear layer.

Baumgartner and Cuenin [45] also observed that ultrasonically energized NaOCl, even at full strength, did not remove the smear layer from root canal walls. Guerisoli *et al.* [46] evaluated the use of ultrasonic energy to remove the smear layer and found it necessary to use 15% ethylenediaminetetraacetic acid (EDTA) with either distilled water or 1% NaOCl to achieve the desired result. 

Mozo *et al.* [47] showed that ultrasonic activation of the irrigation with Irrisafe tips was the most effective procedure for eliminating the debris and opening up the dentinal tubules, especially in the apical third. Mancini *et al.* [48] showed that none of the tested activation/delivery systems (EndoActivator, EndoVac, and passive ultrasonic irrigation) completely removed the smear layer from the dentinal walls. Andrabi *et al.* [49] compared the effect of PUI with manual dynamic irrigation on smear layer removal from root canals using a closed apex *in vitro* model. Findings showed that both activation techniques are important adjuncts in removing the smear layer.

Curtis and Sedgley [50] showed that final irrigation with the VSS (an ultrasonic irrigation device) compared with conventional needle irrigation delivery resulted in significantly less debris present in root canals at 1 and 3-mm distances from the WL.

Kocani *et al.* [51] showed that ultrasonic and manual instrumentation of the root canal and irrigation with combined solutions was effective in removal of the smear layer from the instrumented walls of the root canal. Al-Ali *et al.* [52] showed that PUI was effective with significantly less remaining smear layer and debris than manual agitation and irrigation with H_2_O_2_. Superiority of ultrasonication of the intra-canal irrigant over the manual technique in removing the smear layer was demonstrated by Ribeiro *et al.* [53]. 

Blank-Goncalves *et al.* [54] showed that sonic and ultrasonic irrigation resulted in better removal of the smear layer in the apical third of curved root canals than conventional irrigation. According to Rodig *et al.* [55] ultrasonic activation of NaOCl and EDTA did not enhance debris removal in curved canals but resulted in significantly more effective smear layer removal at coronal levels.

Paque *et al.* [56] confirmed the efficacy of ultrasonic activation of NaOCl and EDTA in removing hard tissue debris. De Moor *et al.* [57] assessed the efficacy of laser activated irrigation (LAI) with erbium: yttrium-aluminum-garnet (Er:YAG) and erbium, chromium: yttrium-scandium-gallium-garnet laser (Er,Cr:YSGG) compared with PUI. Findings revealed that LAI techniques using erbium lasers (Er:YAG or Er,Cr:YSGG) for 20 sec are as efficient as PUI with the intermittent flush technique.

***Ultrasonics vs. sonic irrigation***

Sonic instruments use a lower frequency (1000-6000 Hz) compared to ultrasonic instruments (25000 Hz). In both types of instruments the file is connected at an angle of 60-90 degrees to the long axis of the handpiece. However, the vibration pattern of ultrasonic files is different from that of sonic instruments. Ultrasonically activated files have numerous nodes and antinodes across the length of the instrument, whereas sonic files have a single node near the attachment of the file and one antinode at the tip of the instrument. Sonic instruments produce an elliptic, lateral movement, similar to that of ultrasonic files [11, 17].

Cameron [39] reported the elevated intracanal temperature from 37 to 45^°^C (in areas close to the tip of the instrument) and 37^°^C (away from the tip) when the irrigant was ultrasonically activated for 30 sec without replenishment. A cooling effect from 37^°^C to 29^°^C was recorded when the irrigant was replenished with a continuous flow of irrigant. The temperature of the irrigant was 25^°^C. The external temperature stabilized at 32^°^C during a continuous flow of the irrigant and reached a maximum of 40^°^C in 30 sec without continuous flow. Ahmad [58] reported a mean 0.6^°^C-rise of temperature during a continuous flow of irrigant. The initial temperature of the irrigant was 20^°^C. A rise of temperature within these ranges will not cause pathological temperature rises in the periodontal ligament.

## Conclusion

*1.* Superiority of ultrasonic irrigation with NaOCl over passive irrigation with syringe is still controversial.

*2.* Superiority of ultrasonic activation of NaOCl on endodontic biofilms over other irrigation methods is controversial.

*3.* Superiority of ultrasonic activation of NaOCl on smear layer removal is controversial. 
